# Pediatric cancer immunotherapy: The moment has arrived

**DOI:** 10.1016/j.omton.2026.201256

**Published:** 2026-09-01

**Authors:** Nirali N. Shah

**Affiliations:** 1 Pediatric Oncology Branch, Center for Cancer Research, National Cancer Institute, National Institutes of Health, Bethesda, MD, USA

## Main text

In 2012, I had the distinct honor of being part of one of the trailblazing teams leading first-in-human/first-in-child chimeric antigen receptor (CAR) T cell trials in B cell acute lymphoblastic leukemia (B-ALL). Little did I know then that we were only at the tip of the iceberg for use of immunotherapy in pediatric oncology. While the multiple CAR T cell approvals for B cell malignancies, including in pediatric B-ALL, reflect the curative potential in relapsed/refractory disease, what has additionally transpired is a cascade of novel immune-effector cell therapy-based approaches, each iteratively building upon the prior to further extend the therapeutic index of immunotherapy.

Highlighted within this timely collection are the many catalysts driving continued progress in pediatric immuno-oncology. Through a robust compilation of original research and review articles, we highlight a broad spectrum of immunotherapeutic strategies utilizing various immune-effector cell types or augmentation approaches across a host of pediatric cancer diagnosis. Leveraging expertise from teams comprising leaders and rising stars in the field, this collection aims to capture the breadth and depth of the momentum of this rapidly evolving field.

The foundation of this collection is embedded across several review articles, which broadly capture the current state-of-the art for advancements of targeted therapies for rare childhood cancers. This includes an overview of the CAR T cell landscape in B-ALL, acute myeloid leukemia (AML), and central nervous system (CNS) tumors. Delving deeper into advancing CAR T cell efforts in CNS tumors, research efforts highlighting prostate-specific membrane antigen (PSMA) targeting in rhabdoid tumors, glypican 2 (GPC2) targeting in medulloblastoma, and boosting CAR T cell functionality in pediatric diffuse midline gliomas are among those included in this collection. Along these lines, efforts in enhancing immunotherapy efficacy by triggering antitumor CD4^+^ and CD8^+^ T cell responses in pediatric cancers, using mRNA-lipid nanoparticle-based vaccine to enhance CAR T cell functionality, further exploring the role of exosomes in pediatric solid tumors, and facilitating disruption of the blood-brain barrier to enhance CNS tumor therapies are among some of the most unique approaches highlighted.

Specific to rare pediatric solid tumors such as osteosarcoma and neuroblastoma where immunotherapy efforts are still being optimized, this collection further explores innovative strategies primed for enhancing antitumor efficacy. In this regard, a shift to alternate cell therapies, including an overview of natural killer (NK) cell-based approaches in pediatric solid tumors and mechanisms to enhance this strategy, including use of CAR NK cells and/or with cytokine support, provide further insights into innovative strategies to harness the immune system. Finally, the collection would not be complete without a discussion on how to optimally enhance outcomes after the original form of cell therapy—hematopoietic stem cell transplantation.

Collectively, this collection highlights transformative strategies that challenge the impossible and redefine how immunotherapy can be leveraged to improve outcomes for patients and families facing a relapsed/refractory and rare cancer diagnosis. I am grateful to all the authors and their teams for their many contributions and continued efforts in advancing the field. As you read these articles, I am certain that you will gain a deeper appreciation for, and share my optimism in, what the future holds for using immunotherapy to improve outcomes in pediatric oncology.

## Acknowledgments

This work was supported in part by the 10.13039/100030692Intramural Research Program, Center of Cancer Research, 10.13039/100000054National Cancer Institute, and 10.13039/100000098NIH Clinical Center, National Institutes of Health (ZIA BC 011823, N.N.S). This research was supported in part by the 10.13039/100030692Intramural Research Program of the National Institutes of Health (NIH). The contributions of the NIH author(s) were made as part of their official duties as NIH federal employees, are in compliance with agency policy requirements, and are considered Works of the United States Government. However, the findings and conclusions presented in this paper are those of the author(s) and do not necessarily reflect the views of the NIH or the US Department of Health and Human Services.

## Declaration of interests

N.N.S. receives research funding from Lentigen, VOR Bio, and CARGO Therapeutics. N.N.S. has also attended advisory board meetings (no honoraria) for VOR, ImmunoACT, and Sobi. N.N.S. received royalties from NIH for the development of CD22 CAR.

